# Distribution and Clinical Characteristics of MIH in Schoolchildren From the Central Peruvian Jungle: A Cross‑Sectional Study

**DOI:** 10.1155/ijod/6626596

**Published:** 2026-01-05

**Authors:** Mayra Belen Barahona-Hernandez, Roxana Patricia López-Ramos, Julio César Sánchez-Sotomayor, Karin Harumi Uchima-Koecklin, Daniel José Blanco-Victorio, Gilmer Torres-Ramos

**Affiliations:** ^1^ Department of Paediatric Stomatology, Faculty of Dentistry, Universidad Nacional Mayor de San Marcos, Lima, Peru, unmsm.edu.pe; ^2^ Postgraduate Department, School of Stomatology, Universidad Científica del Sur, Lima, Peru, cientifica.edu.pe; ^3^ Private Clinic, Avenue Vilcabamba G19 Chorrillos, Lima, 15081, Peru; ^4^ Life Sciences Institute, University of Michigan, Ann Arbor, Michigan, USA, umich.edu; ^5^ Department of Statistics and Demography, Faculty of Science and Engineering, Universidad Peruana Cayetano Heredia, Lima, Peru, upch.edu.pe

**Keywords:** children, developmental defects of enamel, molar hypomineralisation, prevalence

## Abstract

**Background:**

Molar incisor hypomineralisation (MIH) is a developmental enamel defect that predominantly affects first permanent molars and frequently involves incisors. However, evidence on MIH prevalence and clinical presentation in jungle regions is limited.

**Aim:**

To determine the distribution and clinical patterns of MIH among schoolchildren in the Central Jungle region of Peru.

**Materials and Methods:**

This cross‐sectional study included 1500 schoolchildren aged 6–12 years from two public schools in Peru’s Central Jungle region, recruited through stratified random sampling. Two calibrated examiners diagnosed MIH using European Academy of Paediatric Dentistry (EAPD) criteria (inter‐examiner *κ* = [0.87]; intra‐examiner *κ* = [0.89]). Categorical variables were analysed using Pearson’s chi‐squared and Fisher’s exact tests (*α* = 0.05, 95% CI).

**Results:**

MIH prevalence was 18.8% (95% CI: [17.0–20.0]). First permanent molars were predominantly affected (upper: 84.8%, 285/336; lower: 90.9%, 288/317) compared to incisors. Pattern I distribution occurred in 78.7% of cases, while patterns II/III (21.3%) showed significant male predominance (*p* = 0.008). Molars exhibited significantly greater severity (*p* = 0.001) with white–cream opacities (28.5%) and predominantly Type III lesions, presenting higher post‐eruptive breakdown and atypical caries rates. Incisors displayed mainly mild Type I demarcated opacities. Upper lateral incisors demonstrated left‐sided predominance (*p* = 0.016).

**Conclusion:**

MIH prevalence was 18.8% among schoolchildren in Peru’s Central Jungle region. First permanent molars were predominantly affected (upper: 84.8%; lower: 90.9%) compared to incisors. Pattern I distribution occurred in 78.7% of cases, while patterns II/III (21.3%) showed significant male predominance. Molars exhibited greater clinical severity with white–cream opacities, Type III lesions, and higher rates of post‐eruptive breakdown and atypical caries, whereas incisors presented mainly mild demarcated opacities.

## 1. Introduction

Molar‐incisor hypomineralisation (MIH), introduced by Weerheijm in 2001 [1], describes a systemic condition involving asymmetrical involvement of one or more permanent first molars, with or without affected incisors [1, 2]. Clinical manifestations range from distinct white–yellow or yellow–brown opacities to severe hypomineralised enamel prone to post‐eruptive breakdown [3, 4]. Affected teeth exhibit hypersensitivity to thermal and mechanical stimuli, impairing masticatory function and oral hygiene compliance, thereby elevating caries risk [5–9]. Incisor involvement additionally imposes aesthetic and psychosocial burdens [10].

MIH aetiology remains multifactorial, encompassing pharmacological exposures (antibiotics, anticonvulsants), childhood illnesses, perinatal hypoxia, and hypomineralised second primary molars [4, 11, 12]. Genetic predisposition and epigenetic mechanisms may further contribute to pathogenesis [13, 14]. Global MIH prevalence is estimated at 15.5%, with regional variation: North America 23.9%, Europe 14.3%–14.4%, Africa 10.9%–14.5% and South America 17.1% [15]. In Peru, most research concentrates on coastal and Andean populations (Lima 10% [16]; Puno 19.8% [17]), leaving jungle regions—which differ substantially in environmental exposures, healthcare access, and sociodemographic characteristics [18]—understudied despite representing considerable national territory.

This study aimed to determine MIH distribution and clinical patterns among schoolchildren aged 6–12 years in Peru’s Central Jungle using European Academy of Paediatric Dentistry (EAPD) criteria [1], thereby informing targeted public health interventions (school‐based screening, preventive strategies and capacity‐building for primary‐care teams) in underserved communities.

## 2. Materials and Methods

### 2.1. Ethics Approval and Study Design

This cross‐sectional study received approval from the Institutional Research Ethics Committee of Peru (Registration number: CIEI‐2020‐19). Written informed consent was obtained from parents or caregivers, and assent was secured from all participating children prior to enrollment.

### 2.2. Study Setting

The study was conducted in Pichanaqui district, Central Jungle of Junín Region, Peru—a predominantly agricultural area with over 39,000 inhabitants characterised by coffee production and a mix of urban and rural zones [18]. This location was selected for its representativeness of underserved rural communities in highland‐lowland transition zones, where limited access to specialised paediatric dental care and environmental factors (variable water quality, nutritional challenges) may influence the prevalence of developmental enamel defects like MIH [17].

### 2.3. Sampling Strategy and Sample Size

Two public schools were randomly selected from the complete roster of six public institutions in the Pichanaqui district using random number generation in Microsoft Excel 2019 (Microsoft Corporation, Redmond, WA, USA). Within these schools, participants were recruited via simple random sampling from enrollment lists.

Sample size was calculated using Epidat software version 4.2 (Dirección Xeral de Saúde Pública, Xunta de Galicia, 2016) based on an anticipated MIH prevalence of 19.8% [17], 2.5% precision, 80% statistical power, and 95% confidence level. Accounting for a 20% non‐response rate, the minimum required sample was 977 participants; this was inflated to 1500 to enhance precision and generalizability.

### 2.4. Selection Criteria

Inclusion criteria: children aged 6–12 years [19, 20] in mixed dentition phase with all four first permanent molars and permanent incisors erupted to at least one‐third of crown height [21].

Exclusion criteria: children with severe systemic illnesses, incomplete records, teeth with extensive restorations obscuring enamel surfaces or confounding enamel conditions (amelogenesis imperfecta, severe dental fluorosis or hypoplasia unrelated to MIH) [22–24].

### 2.5. Examiner Training and Calibration

Two paediatric dentistry examiners underwent structured training provided by a senior paediatric dentist with over 20 years of experience (originally trained in Brazil). Training comprised two sessions: (1) practical instruction using standardised images illustrating the spectrum of MIH presentations and other developmental enamel defects; (2) independent assessment of 30 digital images classified according to EAPD criteria.

Reliability was assessed using Cohen’s kappa coefficient: inter‐examiner agreement *κ* = 0.87; intra‐examiner reliability (reassessed after a 10‐day interval to minimise recall bias) *κ* = 0.89—both indicating substantial agreement. Disagreements during calibration were resolved through consensus discussion with the senior trainer. Examiners remained blinded to participants’ demographic and socioeconomic data throughout assessments.

### 2.6. Clinical Examination

Examinations were conducted in school dental offices equipped with dental units and LED lighting. All participants received prophylaxis prior to evaluation. Teeth were assessed in a wet state using an oral mirror and periodontal probe, following EAPD guidelines [1].

### 2.7. Diagnostic Criteria

MIH was diagnosed according to EAPD criteria [1] (Table 1).

**Table 1:** MIH diagnostic classification.

Category	Definition
Distribution pattern [25]	
Pattern I	Molars only
Pattern II	Molars and ≥1 affected incisor
Pattern III	Upper and lower molars and incisors affected
Clinical status [1]	
Demarcated opacity	White–cream or yellow–brown demarcated opacity
Post‐eruptive breakdown	Loss of enamel structure after tooth eruption
Atypical restoration	Restoration not consistent with caries pattern
Atypical caries	Caries in unusual locations associated with MIH
Extraction due to MIH	Tooth extracted because of MIH severity
Not categorized	Unable to classify due to insufficient information
Lesion extension [1]	
Type I	<1/3 of tooth surface affected
Type II	≥1/3 but <2/3 of tooth surface affected
Type III	≥2/3 of tooth surface affected

### 2.8. Statistical Analysis

Data was recorded in Microsoft Excel 2019 and analysed using Stata version 16.0 (StataCorp LLC, College Station, TX, USA). Qualitative variables were described using absolute and relative frequencies. Normality assumptions were assessed prior to analysis.

Inferential comparisons employed chi‐squared tests for categorical variables and Fisher’s exact test when expected cell counts were <5. The difference of proportions test compared to the overall proportions of affected teeth between right and left hemiarcades based on total counts per arch. All tests were two‐tailed with significance set at *p*  < 0.05; 95% confidence intervals are reported. Participants with missing data were excluded from analysis.

## 3. Results

The prevalence of MIH among schoolchildren was 18.8% (95% CI: 17.0%–20.0%). This narrow confidence interval indicates a precise estimate of MIH prevalence in this population (Table 2).

**Table 2:** Distribution of molar incisor hypomineralisation (MIH).

Oral condition	*n* (%)	CI (95%)
Without MIH	1218 (81.2)	
MIH	282 (18.8)	17.0%–20.0%
Total	1500 (100.0)	

*Note:* 95% CI, 95% confidence interval for the prevalence of MIH (17.0%–20.0%).

### 3.1. MIH Distribution

Table 3 shows that MIH predominantly affects the first permanent molars. In the upper arch, 285 of the 336 affected teeth were first molars (84.8%), and in the lower arch, 288 of the 317 affected teeth were first molars (90.9%, Table 3). Incisor involvement was considerably less common: central incisors accounted for 44/336 (13.1%) of affected teeth in the upper arch and 25/317 (7.9%) in the lower arch, whereas lateral incisors were uncommon (upper 7/336, 2.1%; lower 4/317, 1.3%). The distribution between the right and left hemi‑arcades was broadly symmetrical. In the upper arch, 176/336 (52.4%) affected teeth were on the right, 160/336 (47.6%) were on the left (no difference in proportions, *p* = 0.198), and in the lower arch, 157/317 (49.5%) were on the right, and 160/317 (50.5%) were on the left (no difference in proportions, *p* = 0.187). The overall comparison between the right and left hemi‑arcades was not statistically significant (*p* = 0.200). The only statistically significant lateral asymmetry observed was for upper lateral incisors: all seven affected upper lateral incisors were on the left side (*p* = 0.016). No significant difference was found for the lower lateral incisors (*p* = 1.000).

**Table 3:** Distribution of teeth affected by molar incisor hypomineralisation (MIH) according to arch and hemiarcades.

Teeth affected by MIH	Upper arch			*p*	Lower arch			*p*
	Right hermiarcade *n* (%)	Left hermiarcade *n* (%)	Total *n* (%)		Right hermiarcade *n* (%)	Left hermiarcade *n* (%)	Total *n* (%)	
Central incisor	24 (7.1)	20 (5.9)	44 (13.1)	0.016 ^∗^	12 (3.8)	13 (4.2)	25 (7.9)	1.000 ^∗^
Lateral incisor	0 (0.0)	7 (2.1)	7 (2.1)		2 (0.6)	2 (0.6)	4 (1.3)	
First molar	152 (45.3)	133 (39.6)	285 (84.8)		143 (45.1)	145 (45.7)	288 (90.9)	
Total *n* (%)	176 (52.4)	160 (47.6)	336 (100.0)		157 (49.5)	160 (50.5)	317 (100.0%)	
Proportion			0.198				0.187	0.200 ^∗∗^

^∗^Fisher’s exact test, used to assess the statistical significance of differences in the distribution of affected teeth between right and left hemicades within each arch;  ^∗^ *p*  < 0.05 denotes statistical significance.

^∗∗^Difference of proportions test, applied to compare the overall proportions of affected teeth between right and left hemicades based on the total counts per arch.  ^∗∗^ *p*  < 0.05 indicates statistical significance.

### 3.2. MIH Pattern

Overall, Pattern I predominated, affecting 222 of 282 children (78.7%), whereas Pattern II/III were present in 60 children (21.3%). By sex, 105/145 males (72.4%) and 117/137 females (85.4%) exhibited Pattern I. Conversely, patterns II/III occurred in 40/145 males (27.6%) compared with 20/137 females (14.6%). The association between sex and the MIH pattern distribution was statistically significant (*p* = 0.008). Specifically, males were more likely than females to exhibit patterns II/III, with 40 males (14.2%) compared to 20 females (7.1%) affected, as detailed in Table 4.

**Table 4:** Distribution of the molar incisor hypomineralisation (MIH) pattern according to sex in schoolchildren aged 6–12 years.

Sex	Pattern			*p* ^∗^
	Pattern I *n* (%)	Pattern II/Pattern III*n* (%)	Total *n* (%)	
Male	105 (37.2)	40 (14.2)	145 (51.4)	0.008
Female	117 (41.5)	20 (7.1)	137 (48.5)	
Total *n* (%)	222 (78.7)	60 (21.3)	282 (100.0%)	

^∗^Chi‐square test, *x* ^2^(2) = 7.1, with 2° of freedom; *p*  < 0.05 indicates a statistically significant difference.

### 3.3. Clinical Characteristics

The clinical pattern of MIH differed significantly between incisors and molars (*p* = 0.001). Although demarcated opacities were common in both groups, molars more frequently presented white–cream (188/576; 28.5%) and yellow–brown opacities (108/576; 16.9%) and were disproportionately affected by more severe outcomes: loss of structure (16/576; 2.4%), atypical restorations (66/576; 10.0%), atypical caries (159/576; 24.1%) and extractions attributed to MIH (10/576; 1.5%). In contrast, incisors presented mainly as demarcated opacities (49/83 white–cream, 28/83 yellow–brown), with very few restorations or carious lesions recorded. (Table 5).

**Table 5:** Distribution of the clinical status of molar incisor hypomineralisation (MIH).

Teeth affected by MIH	Incisors	Molars	*p*
Clinical status	*n* (%)	*n* (%)	
Demarcated white–cream opacity	49 (7.4)	188 (28.5)	0.001 ^∗^
Demarcated yellow–brown opacity	28 (4.3)	108 (16.9)	
Loss of structure	4 (0.6)	16 (2.4)	
Atypical restoration	2 (0.3)	66 (10.0)	
Atypical caries	0 (0.0)	159 (24.1)	
Extracted due to MIH	0 (0.0)	10 (1.5)	
Not categorised	0 (0.0)	29 (4.4)	
Total *n* (%)	83 (12.6)	576 (87.4)	

^∗^Fisher’s exact test; *p*  < 0.05 indicates a statistically significant difference.

### 3.4. Extension of Lesions

The extent of MIH differed by tooth type: molars most frequently presented with extensive lesions (Type III: upper 133/285, 46.7%; lower 144/288, 49.5%) and showed a significantly different distribution between arches (*p* = 0.001), whereas incisors predominantly presented less extensive defects (Type I) with no significant arch‑related difference (*p* = 0.645) (Table 6).

**Table 6:** Distribution of molar incisor hypomineralisation according to extension.

Affected teeth	Upper molars		Lower molars				Upper incisors		Lower incisors		
Extension	*n*	%	*n*	%	*p* ^∗^		*n*	%	*n*	%	*p* ^∗∗^
Type I	35	12.3	24	8.8	0.001		24	43.6	12	41.4	0.645
Type II	117	41.1	120	41.7			20	39.2	15	51.7	
Type III	133	46.7	144	49.5			7	12.8	2	6.9	
Total	285	100.0	288	100.0			51	100.0	29	100.0	

^∗^Chi‐square test for upper and lower molars, *x* ^2^ (2) = 14.46, with 2° of freedom; *p* < 0.05 significant.

^∗∗^Fisher’s exact test for upper and lower incisors, *p* = 0.001, *p* greater than 0.05 not significant.

### 3.5. Distribution of Lesion Extent and Clinical Features of MIH by Tooth (Incisors and First Molars)

**Figure 1:**
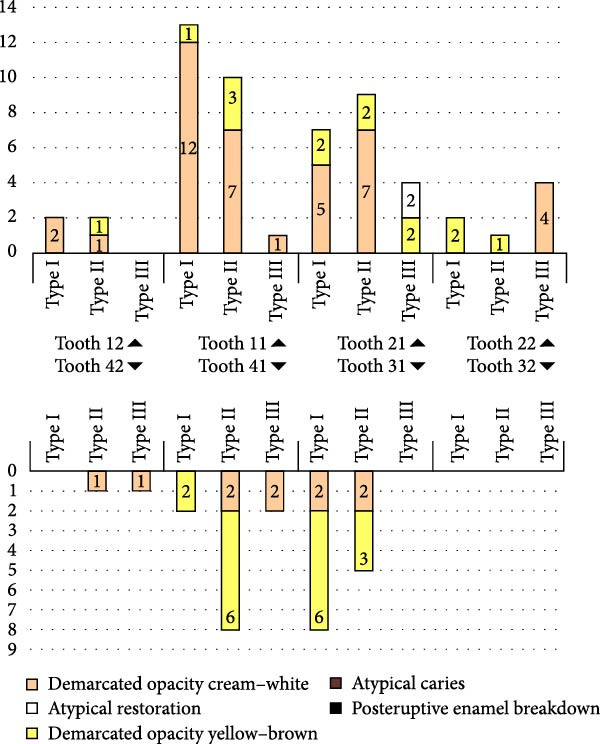
Distribution of lesion extent (Types I–III) and clinical features of MIH by incisor type. No significant difference in lesion extent was observed between upper and lower incisors (*χ*²(2) = 0.89, *p* = 0.645).

Figure 1 illustrates the distribution of lesion extent (Types I–III) and clinical features of MIH across incisor types. Upper central incisors (teeth 11 and 21) showed the highest frequency of involvement, mainly with Type I and II defects. Demarcated opacities, particularly cream–white, were the predominant clinical feature, whereas post‑eruptive enamel breakdown, atypical restorations and atypical caries were rare. Consistent with Table 6, no statistically significant difference in lesion extent was observed between upper and lower incisors (*χ* ^2^(2) = 0.89, *p* = 0.645).

**Figure 2:**
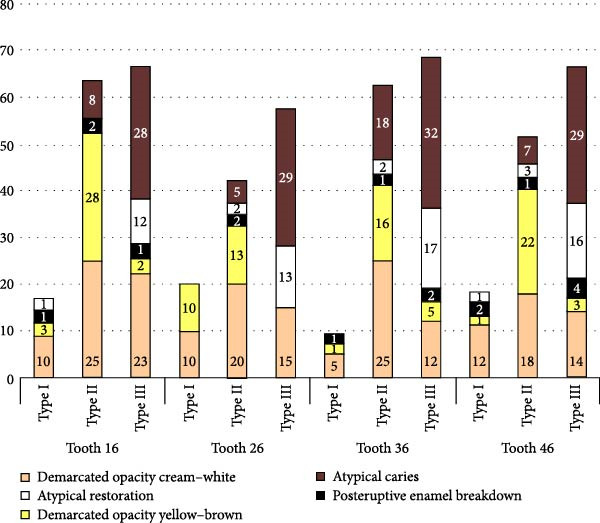
Distribution of lesion extent (Types I–III) and clinical features of MIH in upper and lower first permanent molars. Lesion extent differed significantly between upper and lower molars, with more extensive defects (Type III) in the lower arch (*χ*²(2) = 14.46, *p* = 0.001).

Figure 2 shows the distribution of lesion extent and clinical characteristics in upper (teeth 16 and 26) and lower (teeth 36 and 46) first permanent molars. Demarcated opacities (cream–white and yellow–brown) were frequent in all molars but were especially common in Type II and Type III lesions. Post‑eruptive enamel breakdown and atypical caries concentrated in the most severely affected teeth, particularly teeth 26, 36 and 46, whereas atypical restorations were less frequent. In agreement with Table 6, the extent of MIH differed significantly between upper and lower molars (*χ* ^2^(2) = 14.46, *p* = 0.001), with lower molars showing a higher proportion of extensive defects (Type III).

## 4. Discussion

This cross‐sectional study established an 18.8% MIH prevalence among children aged 6–12 years in Central Jungle, Peru—consistent with global estimates (one in six to seven children affected) [15, 26–28] and Latin American reports from Peru (15.9%–20.2%) [16, 17]. Notable sex‐related differences emerged: patterns II/III predominated in males, whereas pattern I was more common in females. These findings contrast with studies reporting no sex predilection [22, 29, 30], but align with research linking MIH severity variations to biological variability or differential environmental exposures during amelogenesis [11, 12]. First permanent molars were predominantly affected, exhibiting extensive lesions, post‐eruptive breakdown, atypical caries and restorations, while incisors presented primarily mild demarcated opacities. A symmetrical distribution across hemiarcades was observed, except for upper lateral incisors (*p* = 0.016), though absolute numbers remained small. These patterns reflect MIH’s heterogeneous nature across tooth types [31, 32], likely influenced by multifactorial genetic and systemic aetiologies [12, 14].

The observed molar involvement (84.8% upper; 90.9% lower) aligns with established literature documenting preferential first molar affectation [1, 22, 33]. Molars predominantly exhibited moderate‐to‐severe defects (Types II/III) characterised by yellow–brown opacities, enamel breakdown, and atypical restorations, whereas incisors displayed mainly Type I lesions (cream‐white opacities) [3, 34]. The high proportion of atypical caries in hypomineralised molars corroborates evidence that defective enamel facilitates rapid breakdown, increased plaque accumulation and dentine exposure [5, 28, 35], complicating restorative management and heightening extraction risk [36]. Hypersensitivity may further reduce oral hygiene tolerance, compounding caries development [6, 8].

MIH represents a significant public health concern in the region, given its association with increased caries susceptibility, hypersensitivity and diminished quality of life [8–10, 31]. The study’s focus on Pichanaqui—a district representing underserved Amazonian communities with limited specialised dental care and potential environmental risk factors (variable water quality and nutritional challenges)—addresses critical gaps in understanding MIH prevalence in ecologically and socioeconomically diverse highland‐lowland transition zones [18]. Early diagnosis is imperative: adoption of standardised diagnostic criteria (e.g., EAPD guidelines) and comprehensive training protocols enhances reporting consistency and facilitate case identification [1, 37, 38]. Preventive strategies—including remineralisation therapies, desensitising agents and minimally invasive interventions—should be promptly implemented to mitigate enamel breakdown risk [38, 39].

For clinicians, integrating routine screening protocols with sealant application and desensitising agents can prevent caries progression in molars. Researchers should investigate hypomineralisation in second primary molars as early MIH predictors [40, 41], explore correlations between MIH and the DMFT index, and evaluate interventions such as fluoride, CPP‐ACP and biomimetic hydroxyapatite supplementation [42–44]. Policymakers must prioritise community‐based oral health programmes in rural areas—including mobile dental units, training for local health workers and referral systems for complex cases—to address regional MIH burden effectively. Raising awareness among parents, teachers and clinicians remains essential for early detection and intervention.

The cross‐sectional design precludes causal inference, and findings may have limited generalisability beyond the specific regional population studied. Nonetheless, the robust sample size (*n* = 1500) and rigorous clinical examinations enabled comprehensive MIH characterisation, enhancing reliability and international comparability. Future longitudinal studies incorporating age stratification and environmental exposure assessments would strengthen causal understanding and inform targeted interventions in similar underserved settings.

## 5. Conclusions

MIH prevalence among schoolchildren in Peru’s Central Jungle was 18.8%, predominantly affecting first permanent molars (84.8% upper; 90.9% lower) with less frequent incisor involvement. Pattern I distribution was most common (78.7%), while patterns II/III (21.3%) showed significant male predominance. Molars exhibited greater clinical severity with white–cream opacities, Type III lesions and higher rates of post‐eruptive breakdown and atypical caries, whereas incisors presented mainly mild demarcated opacities. These findings underscore MIH as a significant public health concern in underserved Amazonian communities, warranting enhanced surveillance and targeted preventive strategies in similar highland‐lowland transition zones.

## Consent

Informed consent was obtained from all study participants.

## Disclosure

All the authors have read and approved the final manuscript.

## Conflicts of Interest

The authors declare no conflicts of interest.

## Author Contributions

Mayra Belen Barahona‐Hernandez, Gilmer Torres‐Ramos and Daniel José Blanco‐Victorio conceived the ideas. Mayra Belen Barahona‐Hernandez and Julio César Sánchez‐Sotomayor and collected the data. Daniel José Blanco‐Victorio, Roxana Patricia López‐Ramos, Karin Harumi Uchima‐Koecklin and Gilmer Torres‐Ramos analysed the data. Mayra Belen Barahona‐Hernandez, Daniel José Blanco‐Victorio and Roxana Patricia López‐Ramos involved in reviewing and editing the manuscript.

## Funding

This study was not funded.

## Data Availability

The data that support the findings of this study are available upon request from the corresponding author. The data are not publicly available due to privacy or ethical restrictions.
